# Preoperative Guidelines for Coronary Artery Bypass Grafting for Health Education and Anxiety Management: Scoping Review

**DOI:** 10.1155/sci5/6688242

**Published:** 2025-11-06

**Authors:** Naryllenne Maciel de Araújo, Silmara de Oliveira Silva, Bruna Vilar Soares da Silva, Maria Améllia Lopes Cabral, Jucielly Ferreira da Fonseca, Roberta Paolli de Paiva Oliveira Arruda Camara, Maria Carolina Batista da Silva, Rodrigo Assis Neves Dantas, Daniele Vieira Dantas

**Affiliations:** ^1^Graduate Program in Nursing, Federal University of Rio Grande do Norte (UFRN), Natal, Rio Grande do Norte, Brazil; ^2^Department of Nursing, Federal University of Rio Grande do Norte (UFRN), Natal, Rio Grande do Norte, Brazil

**Keywords:** anxiety, health education, myocardial revascularization, nursing, preoperative period

## Abstract

**Introduction:**

Patients in the preoperative period for coronary artery bypass grafting (CABG) frequently experience significant anxiety and depression, in addition to the physical symptoms necessitating the procedure. Preoperative health education is a crucial intervention to enhance patient knowledge, manage this psychological distress, and improve preparedness for the surgical process.

**Objective:**

This scoping review aims to map the guidelines and strategies used for patient health education and anxiety management in the preoperative period for CABG.

**Method:**

A scoping review was conducted in March 2024, adhering to the JBI framework. Thirteen databases were searched with no temporal or linguistic restrictions. Eligibility criteria were limited to open-access studies focused on preoperative interventions for CABG. The findings were analyzed and synthesized descriptively.

**Results:**

The final selection included 12 studies. Key educational guidelines identified focused on surgical planning, detailed explanations of the procedure, familiarization with medical devices (e.g., drains and monitors), and postoperative care instructions. The reviewed literature consistently demonstrated that these educational interventions are effective in managing patient anxiety and are associated with a reduction in postoperative complications.

**Conclusion:**

This review provides key guidelines for preoperative education to help clinicians reduce patient anxiety and improve surgical outcomes for those undergoing CABG.

## 1. Introduction

Cardiovascular diseases (CVDs) represent a major global health burden, affecting a large portion of the population, including economically active individuals, and placing significant strain on healthcare systems and national economies [1–3]. A direct consequence of this burden is the rising incidence of myocardial ischemia and acute myocardial infarction (AMI). Treatment decisions for these conditions are based on the patient's hemodynamic stability and the extent of coronary artery impairment, with the primary goal of optimizing postintervention quality of life [4].

Common therapeutic approaches include medication and surgical procedures, with coronary artery bypass grafting (CABG) being a prevalent surgical option [4]. In the United States, approximately 2000 CABG procedures are performed per million inhabitants annually. Similarly, in Brazil, the volume of CABG surgeries has grown over the last decade, now representing 70% of all cardiovascular procedures performed in the country [4, 5].

The preoperative period for CABG is a significant source of psychological distress, often characterized by heightened anxiety, fear, and depression that can negatively impact a patient's physiological stability [6]. This distress is frequently linked to a lack of understanding of the surgical process, including the procedure itself, its objectives, and the subsequent recovery phase [6, 7].

To address this, preoperative guidance delivered by nursing staff as structured health education is a critical intervention. By using accessible, evidence-based educational tools, this approach can effectively alleviate patient anxiety and has been shown to reduce postoperative complications [2, 4]. However, while various educational methods exist—such as booklets, videos, and verbal instructions—they often focus on clinical data like hemodynamic parameters and imaging results. Consequently, these materials may not adequately address the specific informational and emotional needs of patients required to manage preoperative stress [8, 9].

For instance, a prior study [10] demonstrated that a nurse-led educational intervention significantly reduced anxiety in patients both before and after cardiac surgery, highlighting the value of integrating such guidance into routine nursing care.

While existing systematic reviews have examined broad educational strategies for cardiac surgery patients, a specific gap remains in the literature. There is a need to identify and consolidate the core guidelines tailored specifically to managing anxiety and providing health education for patients in the immediate preoperative period for CABG [2, 8, 10].

Therefore, this study is justified by the need to synthesize the primary scientific evidence on patient guidance before CABG. This work aims to support evidence-based decision making in clinical nursing practice and to stimulate further research. Accordingly, the objective of this study is to map the guidance provided to patients in the preoperative period of CABG for the purposes of health education and anxiety management.

## 2. Method

This scoping review was conducted in accordance with the JBI Reviewer's Manual and follows the Preferred Reporting Items for Systematic Reviews and Meta-Analyses extension for Scoping Reviews (PRISMA-ScR) checklist (S1) [11, 12]. The review protocol was designed based on the JBI framework, which includes defining the research question, establishing eligibility criteria, developing a comprehensive search strategy, screening and selecting studies, and synthesizing the evidence [11].

To ensure the originality of the research question and avoid duplication, a preliminary search of international registration platforms was conducted. These included the International Prospective Register of Systematic Reviews (PROSPERO), Open Science Framework (OSF), the Cochrane Library, JBI Evidence Synthesis (formerly COnNECT+), and the Database of Abstracts of Reviews of Effects (DARE). This initial search revealed a scarcity of literature on the topic, confirming the need for this review. The study protocol was subsequently registered on the OSF platform (https://osf.io/dbe7h/).

The research question was formulated using the Population, Concept, and Context (PCC) framework: Population (*P*): patients undergoing CABG; Concept (*C*): guidelines for health education and anxiety management; and Context (*C*): the preoperative period. This framework guided the development of the primary research question: “What guidelines are provided to patients in the preoperative period of CABG for health education and anxiety management?”

A systematic search was performed in March 2024 across 13 databases and platforms: Web of Science, Virtual Health Library (VHL), Scopus, Scientific Electronic Library Online (SciELO), PubMed, Cochrane Library, ScienceDirect, Cumulative Index to Nursing and Allied Health Literature (CINAHL), CAPES Catalog of Theses and Dissertations, Gale Academic OneFile, and Google Scholar. This was supplemented by a gray literature search in ACS Journals and the Open Access Scientific Repositories of Portugal (RCAAP).

The search strategy combined descriptors from Medical Subject Headings (MeSH) and Health Sciences Descriptors (DeCS), such as “Myocardial Revascularization,” “Anxiety,” “Health Education,” “Patient Education as Topic,” and “Preoperative Care.” The search strings were constructed using Boolean operators (“AND”/“OR”) and adapted for the specific syntax of each database.

Database access was facilitated by the CAPES Periodical Portal via the Federated Academic Community (CAFe) provided by the Federal University of Rio Grande do Norte (UFRN). Inclusion criteria were as follows: (1) full-text, open-access publications; (2) studies addressing the research objective; and (3) no language or date restrictions. Exclusion criteria were as follows: abstracts only, letters to the editor, opinion articles, and studies focused on other surgical procedures or that did not align with the research question.

Study selection was a two-stage process conducted by two independent reviewers (MALC and BVSS). First, they screened titles and abstracts for relevance. Second, they performed a full-text review of potentially eligible articles to determine final inclusion. A third reviewer (NMA) was consulted to resolve any disagreements at either stage.

Data from the included studies were extracted, charted, and analyzed using descriptive statistics. The Patterns, Advances, Gaps, Evidence for Practice, and Research Recommendations (PAGER) framework [13] was then used to structure the analysis and reporting of the results, ensuring a comprehensive synthesis of the evidence.

## 3. Results

**Figure 1:**
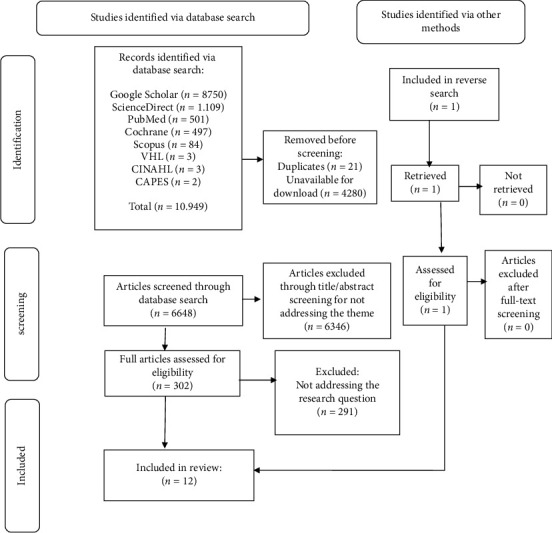
Flowchart adapted from the Preferred Reporting Items for Systematic Reviews and Meta-Analyses extension for Scoping Reviews (PRISMA-ScR). Natal, RN, Brazil, 2024.

The initial search yielded 10,949 studies across all data sources. These included 8750 from Google Scholar, 1109 from ScienceDirect, 501 from PubMed, 497 from Cochrane, 84 from Scopus, three each from VHL and CINAHL, two from the CAPES Catalog of Theses and Dissertations, and one from a reverse search. No results were retrieved from the remaining sources. After screening titles and abstracts against the eligibility criteria, 12 articles were selected for the final sample.  Figure 1 provides a complete flowchart of the identification, screening, and inclusion process.

The selected studies originated from several countries, primarily Iran (four publications, 33%) and Canada (two publications, 17%). Brazil, Pakistan, Lebanon, the United Kingdom, the European Union, and the United States each contributed one publication (9%). Publication years ranged from 2000 to 2022. Regarding study design, the sample included seven randomized clinical trials (59%), two systematic reviews without meta-analysis (17%), one quasi-experimental study, one systematic review with meta-analysis, and one expert guideline (9% each). These data are summarized in Table 1.

Table 2 presents the main preoperative guidelines identified in the review. The most frequent topics were postoperative recovery and rehabilitation (*n* = 8), followed by explanations of the surgical procedure and its purpose (*n* = 7).

Verbal instructions were the primary educational strategy, employed in all included studies. Videos were also used in three publications (25%), while other approaches like booklets, leaflets, and telephone support were each reported once (9%). Health education was a universal strategy for anxiety management across all studies. Three of these studies (25%) also incorporated emotional and family support as key interventions. Finally, the findings were synthesized and organized using the PAGER framework. This structure identifies key PAGER from the literature, as detailed in Table 3.

## 4. Discussion

The diverse locations and publication years of the included studies highlight the global relevance of this topic and the need for ongoing research. Moreover, the high quality of the selected articles provides robust, evidence-based support for nursing practice in the preoperative care of CABG patients.

Preoperative health education is a dynamic process that can incorporate various strategies, from verbal guidance and group lectures to videos, booklets, and telephone support [2, 8, 14, 16]. The specific guidelines that should inform these strategies are outlined in Table 3. Key topics include explanations of the CABG procedure, perioperative care, pain management, rehabilitation exercises, lifestyle adjustments, and emotional support [2, 8, 14–16, 20]. This aligns with broader literature indicating that comprehensive guidance is essential for improving postoperative quality of life [9, 21, 22].

The guidelines identified in this review are consistent with interventions validated by the Nursing Intervention Classification (NIC). For instance, one study demonstrated that applying a NIC-guided educational plan yielded effective results in preparing patients for CABG [14].

Verbal instruction was the most common educational method found in this review. This prevalence may be due to practical reasons, such as its utility for patients with low literacy who cannot read leaflets or manuals [17, 18, 23]. However, it may also reflect a need for professionals to develop skills in using other educational technologies [21]. Audiovisual resources, for example, can be particularly helpful for patients with hearing or reading difficulties [22].

Across all studies, health education was the primary intervention for managing patient anxiety. Its efficacy was demonstrated through improved patient knowledge and reduced levels of anxiety, depression, and stress, which can contribute to shorter hospital stays [8, 15, 16, 20]. The central role of nursing in this process is clear. Studies led by nurses that used educational technologies and nonpharmacological strategies showed significant improvements in patient understanding [8, 9] and a measurable reduction in anxiety [8, 9, 24–26] and pain [27], which was sustained long term.

Beyond reducing postoperative complications and length of stay [2, 14, 15, 22], nursing-led health education plays a crucial role. By using preoperative guidelines, nurses facilitate a learning process that empowers patients and families to cope with hospitalization and promotes effective communication within the healthcare team [7, 8].

This review also identified several gaps that suggest opportunities for future research. There is a need for more effective methodologies to evaluate educational interventions, as well as long-term follow-up to assess their impact on postoperative quality of life [4, 14, 16–20]. As highlighted in a prior study [21], there is a particular lack of comparative studies evaluating the effectiveness of different educational techniques. Given the diversity of available resources, such research would be highly beneficial.

### 4.1. Limitations

The main limitation of this study is its reliance on open-access literature, which may have restricted the number of included articles. Additionally, by focusing solely on cardiac surgery, the findings may have limited generalizability to other surgical populations.

## 5. Conclusion

This review successfully mapped the key guidelines for the preoperative education of patients undergoing CABG. The evidence confirms that these guidelines, when delivered as structured health education, are effective for managing patient anxiety. This, in turn, helps reduce postoperative complications that can arise from a lack of patient awareness or understanding.

Furthermore, this study highlights the variety of educational tools and techniques available to support healthcare professionals. The findings reinforce the need to implement evidence-based preoperative programs that apply this knowledge, ultimately improving patient care and surgical outcomes.

## Figures and Tables

**Table 1:** Summary of studies by ID, author, country, year, objective, design, and sample.

ID	Author	Country/year	Objective	Design/sample
S1	Araújo et al. [8]	Brazil/2022	To evaluate the effect of the audiovisual resource on the acquisition of knowledge about the level of anxiety in the preoperative period of CABG.	Randomized clinical trial/44 patients
S2	Ali et al. [14]	Pakistan/2021	To identify the effect of nurse-led preoperative education on minimizing the level of anxiety among patients awaiting myocardial revascularization surgery.	Randomized clinical trial/136 patients
S3	Neumann et al. [4]	European Union/2019	To support the treatment of patients with coronary artery disease through pragmatic, evidence-based recommendations.	Expert guideline
S4	Khajian Gelogahi et al. [15]	Iran/2018	To determine the effect of the intentional presence of a holistic nurse on anxiety, stress, and depression in patients undergoing CABG.	Randomized clinical trial/80 patients
S5	Babaee et al. [16]	Iran/2007	To assess the ability of the health education program to improve health quality of life of patients with myocardial revascularization surgery.	Randomized clinical trial/70 patients
S6	Deyirmenjian et al. [17]	Lebanon/2006	To evaluate the impact of preoperative patient education on the anxiety and recovery of Lebanese patients undergoing open heart surgery.	Quasi-experimental study/110
S7	Shuldham et al. [18]	United Kingdom/2002	To elucidate the consequences of preoperative education, given before hospitalization, on postoperative pain, anxiety, depression, and well-being in the 6 months after a first episode of coronary artery surgery.	Randomized clinical trial/356 patients
S8	Shuldham [19]	USA/2001	To establish whether preoperative education benefits patients after coronary artery bypass surgery and identify affected outcomes.	Systematic review with meta-analysis/10 studies
S9	Arthur et al. [20]	Canada/2000	To examine the effect of a multidimensional preoperative intervention on preoperative and postoperative outcomes in low-risk patients awaiting elective coronary artery bypass grafting	Randomized clinical trial/249 patients
S10	Shirdel et al. [21]	Iran/2020	To provide adequate preoperative supportive conditions and improve anxiety and vital signs for patients undergoing CABG	Randomized clinical trial
S11	Abdelrahman [10]	Iran/2021	To examine the impact of nurse-led preoperative and postoperative educational interventions on reducing anxiety levels in patients undergoing cardiac surgery.	Systematic review
S12	Shi et al. [2]	Canada/2022	To evaluate the effectiveness of patient education for secondary prevention related to behavior changes and risk factor modifications on psychological outcomes in patients with coronary heart disease.	Systematic review

*Note:* Natal, RN, Brazil, 2024.

**Table 2:** CABG preoperative guidelines recommended in the studies.

Studies	Preoperative guidelines
S1; S2; S3; S4; S5; S6; S7; S8; S10; S11	Recovery and rehabilitation after surgery: Planning daily activities and work postsurgery.
S1; S3; S4; S5; S7; S8; S9; S10; S11	Understanding the meaning and approach of CABG surgery: Explanation of the procedure type.
S1; S4; S6; S7; S8; S9; S10; S11; S12	Immediate postoperative period: Introduction to the intensive care unit and its routines; orientation regarding the patient's physical environment, including the bed, equipment, and devices to be used, equipment and devices that will be used on the patient; guidance on early ambulation; explanation of the discharge process and postdischarge routine.
S1; S4; S6; S8; S9	Intraoperative procedures: Explanation of devices used, including ventilators, drains, and monitoring equipment; overview of required anesthesia.
S3; S5; S6; S7; S8; S10; S11; S12	Impact on quality of life: Discussion on hospitalization restrictions and potential consequences for the patient's life; exploration of risks and benefits associated with the CABG procedure.
S3; S4; S7; S8; S9; S10	Understanding the multidisciplinary team: Introduction to team members and their respective roles.
S4; S6; S7; S8; S9; S10; S11; S12	Patient admission information: Overview of institution routines, visitation protocols, and admission procedures.
S2; S5; S7; S8	Education based on the International Nursing Classification (NIC) in the physiological domain: Management of activities and exercises; nutritional support and promoting physical comfort; pain management strategies.
S3; S4; S5; S6	Pain management
S5; S6; S7; S8; S10; S11; S12	Application and explanation of exercises and physical activities: Demonstration and explanation of breathing exercises.
S3; S7; S8; S9; S10; S11	Preoperative routine: Overview of team visits and decision-making processes; explanation of preoperative exams and presurgery preparation protocols; guidelines on personal hygiene procedures.
S4; S5; S7; S10	Explanation of the purpose of hospitalization and anticipated length of stay.
S4; S8; S9; S11	Sharing success stories of previous patients who underwent similar procedures.
S2; S8	Education based on the International Nursing Classification (NIC) in the family domain: Support for caregivers and involvement of family members; mobilization of family resources and support systems.
S8; S10	Provision of psychological and emotional support throughout the surgical process

*Note:* Natal, RN, Brazil, 2024.

**Table 3:** Evaluation of study findings using the PAGER structure.

Study pattern	Advances achieved	Existing gaps	Evidence for practice	Recommendations for future research
Health education (S1; S2; S3; S4; S5; S6; S7; S8; S9; S10; S11; S12)	- Audiovisual resources and educational technologies enhance patient comprehension (S1; S8; S11). - Updated guidelines coupled with effective team communication facilitate decision-making processes (S3; S9; S12). - Strategic planning serves as an intervention to augment patient knowledge (S5; S12). - Implementing a health education program from the moment of admission proves beneficial (S7). - Health education conducted with patients leads to improved recovery outcomes (S8; S9; S10; S11). - Diverse techniques and technologies are employed for patient education (S11).	- Need for awareness of the correct use of audiovisual resources prior to application (S1). - Assessment of patients awaiting surgery for extended periods is necessary to enhance data accuracy (S2; S3; S9; S11); - Institutions' unreadiness to implement health education-based planning is lacking (S5). - Generalization of guidelines often overlooks disease severity and individual surgical peculiarities (S6; S7). - Evidence is constrained by study methodology limitations (S8; S10).	- Technological and educational strategies bolster patient education efforts (S1; S8; S11). - The exclusive use of audiovisual resources tailored to specific surgical procedures is recommended (S1; S3; S12). - Preoperative education effectively reduces anxiety levels (S2; S7; S8; S10; S11; S12). - Protocol development should involve a multidisciplinary team (S3; S10). - Health education initiatives should commence before surgery (S3; S10). - The presence of holistic nursing care diminishes stress, anxiety, and depression (S4). - Health education positively impacts post-CABG surgery quality of life and anxiety levels (S5; S8; S11; S12). - Shorter hospital stays are associated with health education interventions (S6; S9; S11). - Preoperative education is best delivered upon admission rather than immediately before surgery (S6; S8). - Anxiety impedes learning outcomes (S6; S10). - Enhanced quality of life is reported postsurgery (S9; S10). - Family support aids in normalizing anxiety levels (S9).	- Refining audiovisual resources using nursing theories improves effectiveness (S1); - Comparative studies across diverse populations yield valuable insights (S1; S7; S8; S9; S10). - Enhancing preoperative guidance through a health education approach is recommended (S2; S8; S11). - Development of new interdisciplinary guidelines is advocated (S3; S9; S10). - Holistic nursing practices are cost-effective and adaptable across various settings (S4). - Booklets and health education techniques are beneficial and easily replicable (S5; S8; S9). - Cultural and educational level adaptations are crucial for effective education delivery (S6; S7; S8; S9). - Enhanced communication skills contribute to improved patient care (S6; S11; S12). - Preoperative physical activity application and its effects warrant exploration (S9).

Contributions to the postoperative period (S8; S11)	- Preoperative health education correlates with more effective recovery (S8; S11).	- Limited patient follow-up hinders data collection and analysis (S8). - Understanding patients' needs is paramount in health education delivery (S11).	- Health education reduces ICU stays and promotes postoperative hemodynamic stability (S8; S11). - Reduced incidence of acute hypertension and enhanced patient satisfaction are observed with health education interventions (S11). - Studies with extended postoperative follow-up periods are necessary (S8). - Disease management–focused education enhances patient outcomes (S11).	- Necessity for studies with extended postoperative follow-up (S8). - Education that concentrates on disease management is warranted (S11).

*Note:* Natal, Rio Grande do Norte, Brazil, 2024.

## Data Availability

All data generated or analyzed during this study are included in this published article.

## Supporting Information

S1. PRISMA-ScR-Fillable-Checklist.

## References

[1] Nascimento B. R., Brant L. C. C., Naback A. D. N. (2022). Carga De Doenças Cardiovasculares Atribuível Aos Fatores De Risco Nos Países De Língua Portuguesa: Dados Do Estudo ‘Global Burden of Disease 2019’. *Arquivos Brasileiros de Cardiologia*.

[2] Shi W., Ghisi G. L. M., Zhang L., Hyun K., Pakosh M., Gallagher R. (2022). A Systematic Review, Meta-Analysis, and Meta-Regression of Patient Education for Secondary Prevention in Patients With Coronary Heart Disease: Impact on Psychological Outcomes. *European Journal of Cardiovascular Nursing*.

[3] Virani S. S., Alonso A., Aparicio H. J. (2021). Heart Disease and Stroke Statistics, 2021 Update A Report From the American Heart Association. *Circulation*.

[4] Neumann F. J., Sousa-Uva M., Ahlsson A. (2019). 2018 ESC/EACTS Guidelines on Myocardial Revascularization. *European Heart Journal*.

[5] Alencar M. d. F. B. d., Cardoso S. d. B., Oliveira A. D. d. S., Santos A. M. R., Cronemberger J. V. B. V., Silva Filho P. S. d. P. (2020). Risco Para Mortalidade Em Pré-Operatório De Revascularização Do Miocárdio. *Research, Society and Development*.

[6] Martins L. M., Kazitani B. S., Bolela F., Maier S. R. d. O., Dessotte C. A. M. (2021). Sintomas De Ansiedade, Depressão E Ansiedade Cardíaca Pré-Operatórios Segundo O Tipo De Cirurgia Cardíaca. *REME-Revista Mineira de Enfermagem*.

[7] Benevides L. M. B., Fernandes L. M., Silva L. d. F. d., Farias M. S., Rabelo A. C. S., Oliveira S. C. (2020). Prática Clínica De Enfermagem Para a Redução Da Ansiedade Em Pacientes No Pré-Operatório Cardíaco: Pesquisa Intervenção. *Online Brazilian Journal of Nursing*.

[8] Araújo N. M., Oliveira E. S., Silva B. V. S., Melo E. B., Dantas R. A. N., Dantas D. V. (2022). Audiovisual Aids in Preoperative Cardiac Surgery Education: A Scoping Review. *Texto Contexto Enferm*.

[9] Silva L. C. d. M. A., Lima Neto A. V., Santos K. V. G. d. (2022). Recomendações Para O Preparo Do Paciente Em Pré-Operatório De Cirurgias Cardíacas: Revisão De Escopo. *Online Brazilian Journal of Nursing*.

[10] Abdelrahman K. H. K. (2021). The Impact of Nurse Led Educational Intervention on Anxiety in Patients Urdergoing Cardiac. *Master of Science in Cardiovascular Nursing*.

[11] Peters M. D. J., Godfrey C., McInerney P., Munn Z., Tricco A. C., Khalil H., Aromataris E., Munn Z. (2020). Scoping Reviews (2020 Version). *Joanna Briggs Institute Reviewer’s Manual*.

[12] Tricco A. C., Lillie E., Zarin W. (2018). PRISMA Extension for Scoping Reviews (PRISMA-ScR): Checklist and Explanation. *Annals of Internal Medicine*.

[13] Bradbury-Jones C., Aveyard H., Herber O. R., Isham L., Taylor J., O’Malley L. (2021). Scoping Reviews: The PAGER Framework for Improving the Quality of Reporting. *International Journal of Social Research Methodology*.

[14] Ali A., Masih S., Rabbi F., Rasheed A. (2021). Effect of Nurse Led Education on Anxiety Level Among Coronary Artery Bypass Grafting Pre-Operative Patients. *Journal of Pakistan Medical Association*.

[15] Khajian Gelogahi Z., Aghebati N., Mazloum S. R., Mohajer S. (2018). Effectiveness of Nurse’s Intentional Presence as a Holistic Modality on Depression, Anxiety, and Stress of Cardiac Surgery Patients. *Holistic Nursing Practice*.

[16] Babaee G., Keshavarz M., Hidarnia A., Shayegan M. (2007). Effect of a Health Education Program on Quality of Life in Patients Undergoing Coronary Artery Bypass Surgery. *Acta Medica Iranica*.

[17] Deyirmenjian M., Karam N., Salameh P. (2006). Preoperative Patient Education for Open-Heart Patients: A Source of Anxiety?. *Patient Education and Counseling*.

[18] Shuldham C. M., Fleming S., Goodman H. (2002). The Impact of Pre-Operative Education on Recovery Following Coronary Artery Bypass Surgery. A Randomized Controlled Clinical Trial. *European Heart Journal*.

[19] Shuldham C. M. (2001). Pre-Operative Education for the Patient Having Coronary Artery Bypass Surgery. *Patient Education and Counseling*.

[20] Arthur H. M., Daniels C., McKelvie R., Hirsh J., Rush B. (2000). Effect of a Preoperative Intervention on Preoperative and Postoperative Outcomes in Low-Risk Patients Awaiting Elective Coronary Artery Bypass Graft Surgery. A Randomized, Controlled Trial. *Annals of Internal Medicine*.

[21] Shirdel Z., Behzad I., Manafi B., Saheb M. (2020). The Interactive Effect of Preoperative Consultation and Operating Room Admission by a Counselor on Anxiety Level and Vital Signs in Patients Undergoing Coronary Artery Bypass Grafting Surgery. A Clinical Trial Study. *Investigación y Educación en Enfermería*.

[22] Lima Neto A. V., Silva I. P. d., Mesquita S. K. d. C. (2023). Application Prototype for Patient Education Before Coronary Artery Bypass Graft Surgery. *Acta Paulista de Enfermagem*.

[23] Barel P. S., Sousa C. S., Poveda V. d B., Turrini R. N. T. (2018). Anxiety and Knowledge of Patients Before Being Subjected to Orthognathic Surgery. *Revista Brasileira de Enfermagem*.

[24] Silva S. d. O., Duarte F. H. d. S., Dutra S. V. O., Ribeiro K. R. B., Dantas R. A. N., Dantas D. V. (2023). Educational Technologies for Caregivers in the Context of Pediatric Oncology Hospital Units: A Scoping Review. *Texto & Contexto-Enfermagem*.

[25] Duarte F. H. S., Silva S. O., Oliveira E. S. (2024). Health Educational Strategies for People Living With HIV: Scoping Review. *Acta Paulista de Enfermagem*.

[26] de Oliveira Silva S., da Silva Duarte F. H., de Souza Costa T. M. (2023). Effectiveness of Multimedia Education for Reducing Anxiety Among Caregivers of Children and Adolescents Undergoing Chemotherapy: Randomized Controlled Trial Protocol. *PLoS One*.

[27] Sarmento S. D., Santos K. V., Dantas J. K., Silva B. V., Dantas D. V., Dantas R. A. (2021). Non-Pharmacological Therapies in the Relief of Cardiac Surgery Postoperative Pain: A Scoping Review. *Online Brazilian Journal of Nursing*.

